# Effects of Animal-Assisted Therapy for Anxiety Reduction in Children and Adolescents: A Systematic Review

**DOI:** 10.3390/jcm14010287

**Published:** 2025-01-06

**Authors:** Constança Brandão, Maria Sampaio, Valéria Sousa-Gomes, Marisalva Fávero, Diana Moreira

**Affiliations:** 1 Institute of Psychology and Neuropsychology of Porto—IPNP Health, 4000-055 Porto, Portugal; cbrandao@ipnp.pt (C.B.); vgomes@umaia.pt (V.S.-G.); dimoreira@ucp.pt (D.M.); 2 Faculty of Psychology and Educational Sciences, University of Porto, 4200-135 Porto, Portugal; mariacoelhosampaio@gmail.com; 3 Social and Behavioral Sciences Department, University of Maia, 4475-690 Maia, Portugal; 4 Center for Psychology, University of Porto—CPUP, 4200-135 Porto, Portugal; 5 Centre for Philosophical and Humanistic Studies, Faculty of Philosophy and Social Sciences, Universidade Católica Portuguesa, 4710-362 Braga, Portugal; 6 Centro de Solidariedade de Braga/Projecto Homem, 4700-024 Braga, Portugal; 7 Laboratory of Neuropsychophysiology, Faculty of Psychology and Educational Sciences, University of Porto, 4200-135 Porto, Portugal; 8 Observatory Permanent Violence and Crime (OPVC), FP-I3ID, Fernando Pessoa University, 4249-004 Porto, Portugal

**Keywords:** animal-assisted therapy, anxiety, anxiety disorders, systematic review, children, adolescents

## Abstract

**Background**: Animal-Assisted Therapy (AAT) is a clinical approach aimed at building valuable human–animal relationships with both preventive and therapeutic goals. It is provided by a healthcare professional and involves animals (meeting certain criteria) as an integral part of the treatment process. This type of therapy has been shown to have multiple benefits in several areas, such as reducing anxiety in a variety of different groups of people. However, few studies have specifically investigated the benefits of AAT in reducing anxiety in children and adolescents. This systematic review aimed to comprehensively assess the evidence regarding the effectiveness of AAT in reducing anxiety among children and adolescents while also identifying research gaps in this field. **Methods**: Studies focusing on the relationship of these two variables were obtained from multiple databases (EBSCO, PubMed, and Web of Science). **Results**: AAT has grown and the literature demonstrates several benefits of this approach. However, few studies have demonstrated the benefits of AAT for reducing anxiety in children and adolescents. **Conclusions**: Most of these few studies show that this type of intervention can help reduce anxiety levels.

## 1. Introduction

Animal-Assisted Therapy (AAT) is a clinical approach aimed at developing a beneficial relationship between humans and animals for preventive and therapeutic purposes [1]. AAT is conducted by a qualified health professional who includes an animal (one that meets specific criteria) as a key element of the therapeutic process. The professional is responsible for setting specific goals for each client and measuring the progress toward those goals [2].

Throughout history, domesticated animals have played a significant therapeutic and supportive role. Primitive drawings of people and wolves sitting around campfires can still be found on cave walls. In Ancient Egypt, many individuals were buried with their beloved pets so they could be together in the afterlife [2]. The therapeutic potential of animal contact was recognized in the 1800s when Florence Nightingale observed that pets could help reduce anxiety levels in institutionalized children and adults [3]. Today, the healing powers of animals are backed by substantial evidence, and they are utilized in various settings, including schools, hospitals, nursing homes, mental health units, physicians’ offices, prisons, and businesses. As professionals gain a better understanding of the benefits of having animals present, therapy dogs are becoming increasingly common as hospital visitors [2].

Research suggests that interacting with animals enhances both physical and emotional well-being. The presence of an animal can facilitate the expression of a range of emotions, including positive feelings like joy and pride, as well as negative ones such as fear. This interaction tends to foster a greater sense of security and comfort [1].

It is important to recognize that the intimacy formed through simple interactions with animals—such as talking to them or petting their fur—can significantly reduce stress levels. This has promising effects on children’s motivation [1]. Therefore, we can conclude that AAT is a therapeutic approach that brings together animals and individuals with specific physical and emotional needs [4].

This type of therapy has been shown to offer several benefits across multiple areas, including improvements in blood pressure, muscle stiffness, and heart rate, e.g., [1,4,5]. It also enhances self-esteem and feelings of security while reducing levels of stress and anxiety. Promising effects have been observed in sensory stimulation, communication, and social interaction [4]. The presence of an animal seems to provide children with a sense of emotional safety, encouraging them to be more active and engaged in relevant activities. Simple tasks, such as speaking to an animal and petting it, help reduce stress and foster creativity, surprise, and humor [1]. Additionally, activities like walking a dog or brushing its fur not only promote physical exercise but also motivate children to take responsibility for their own care [5].

Research indicates that AAT has a significantly positive impact on anxiety. Several studies demonstrate its effects on blood pressure, heart rate, and stress levels, offering varied evidence of the effectiveness of this therapeutic approach [6,7,8].

It is important to note that AAT should not be applied universally to all cases or individuals. Each case has its own specificities and must be considered within its particular context [4].

While there are studies demonstrating how AAT helps reduce anxiety in various populations, e.g., [1,4,5,6,7,8], few specifically examine its benefits for children and adolescents. Therefore, this systematic review aims to provide a comprehensive assessment of the evidence regarding the effectiveness of AAT in reducing anxiety in children and adolescents, while also identifying research gaps in this field.

## 2. Method

As previously mentioned, this systematic review aims to explore how an AAT approach can be used to address anxiety disorders in both childhood and adolescence. To achieve this, we consulted several databases, including EBSCO, PubMed, and Web of Science. To gather the maximum number of studies on this topic, we began with the following search expression: TI (“animal-assisted therapy” OR “animal assisted therapy” OR “pet therapy” OR “pet-oriented therapy” OR “animal therapy” OR “animal intervention” OR “canine assisted psychotherapy” OR AAT OR “animal-assisted psychotherapy” OR “animal assisted psychotherapy” OR “pet psychotherapy” OR “pet-oriented psychotherapy” OR “animal psychotherapy” OR “pet-oriented child psychotherapy”) AND TI (anxiety disorder* OR anxiety OR “generalized anxiety disorder”) AND TI (depressive disorder* OR “depressive symptoms” OR “major depressive disorder” OR MDD).

As the number of articles obtained through this research was insufficient for a systematic review, a new analysis was carried out. The expression used was as follows: AB (“animal-assisted therapy” OR “animal-assisted therapy” OR “pet therapy” OR “pet-oriented therapy” OR “animal therapy” OR “animal intervention” OR “canine assisted psychotherapy” OR AAT OR “animal-assisted psychotherapy” OR “animal assisted psychotherapy” OR “pet psychotherapy” OR “pet-oriented psychotherapy” OR “animal psychotherapy” OR “pet-oriented child psychotherapy”) AND TX (anxiety disorder* OR anxiety OR “generalized anxiety disorder”).

An initial total of 419 articles was identified. The removal of duplicates resulted in 205 articles, published between 1981 and 2021.

The abstracts of the identified articles were independently reviewed by two reviewers based on the following inclusion criteria: (1) the participants of the studies must be children and adolescents (age 18 and under) with anxiety problems; and (2) the studies must involve Animal-Assisted Therapy (AAT). Some articles were excluded for the following reasons: (1) incorrect publication type (theoretical studies, narrative reviews, systematic reviews with or without meta-analyses, and comprehensive reviews); (2) incorrect population (studies where participants were older than 18 or had any psychopathology unrelated to anxiety); and (3) incorrect outcomes (studies that did not focus on the two variables of interest, such as studies which focused on the use of stuffed animals or robots, which does not qualify as AAT).

**Figure 1:**
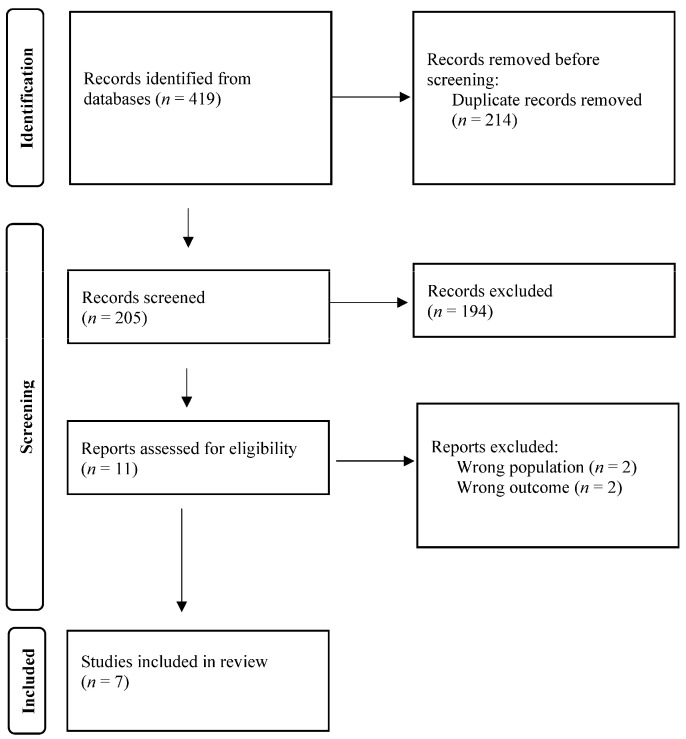
Flowchart of literature review process.

As presented in Figure 1, the independent analysis resulted in 205 articles. The agreement index in the study selection process was evaluated using Cohen’s Kappa, which indicated an almost perfect agreement with K = 0.95, *p* < 0.001 [9]. The reviewers engaged in discussions regarding any disagreements and successfully resolved them through consensus.

After reviewing the articles, 198 were rejected for not meeting the eligibility criteria. Consequently, this systematic review includes a total of seven articles that fulfilled the objectives of this study.

## 3. Results

Out of the seven studies, six compared an animal intervention group to a control group, while only one study did not include a control group. All studies focused on AAT involving dogs. The number of participants in these studies ranged from 15 to 153, with an average of 66 participants (*M* = 66).

The study conducted without a control group involved 21 children who participated in a 20 to 60 min AAT session before undergoing an MRI scan. The duration of interaction during and after the exam varied depending on the length of the scan. The results indicated that 90% of the children were able to complete the MRI, while 10% were unable to proceed. Before the exam, none of the participants reported feeling relaxed or unworried. In fact, 40% indicated they were worried, another 40% described themselves as very worried, and 20% expressed feelings of anger. After the MRI, 55% of the children reported feeling relaxed or not worried. However, 30% remained slightly worried or fairly worried. A comparison of the participants’ pre- and post-intervention Likert scale scores revealed an average decline of 1.65 points in the median score, indicating a significant improvement (Wilcoxon *t*-test, *p* < 0.01). Interestingly, two participants reported feeling angrier after the exam, but both acknowledged that the therapy dog helped them feel more relaxed or less worried. One child was unable to complete the exam, but all participants expressed a desire to have the therapy dog with them during their next visit. Additionally, 83% stated that the dog made them feel more relaxed or less worried after the MRI scan [10]. Of the six studies that used a control group, three did find statistically significant differences between groups, two studies did not find statistically significant differences between groups, and one did not find evidence that anxiety levels were affected by the AAT session.

Among the three studies that identified statistically significant differences between groups, one included 153 children who had all experienced child abuse, aged between 7 and 17 years. The sample comprised 10 males and 143 females. The participants were divided into three groups: Group 1 had no dogs, Group 2 had dogs but no stories, and Group 3 had both dogs and stories. These stories consisted of therapeutic stories about dogs that were specific to the session topics. The group without dogs participated in 12 sessions that covered various topics and activities related to common challenges faced by survivors of sexual abuse. These topics included trust, self-esteem, keeping secrets, triggers, boundaries, emotions, and identifying welcomed versus unwanted touch. Participants in the group with dogs but no stories also participated in 12 sessions with the same structure as the no-dogs group. They enjoyed a 30 min interaction with the dog and its handler in the lobby. They spent an additional 10 to 15 min in the session as part of an introductory activity before leaving the group. The group with dogs and stories followed a similar format to the other groups but incorporated storytelling into the sessions. These stories provided structure and depth to the therapy dog visits. A series of questions about the stories was developed to facilitate a smooth transition from the dog’s visit to the specific discussion topics of the group. Results indicated that children in the dogs-with-stories group reported lower levels of anxiety, depression, anger, PTSD, dissociation, and sexual concerns compared to those in the no-dogs group. Meanwhile, children in the dogs-without-stories group also demonstrated lower levels of depression (*β* = −0.266, *p* < 0.05), anger (*β* = −0.310, *p* < 0.01), and dissociation (*β* = −0.217, *p* < 0.05) compared to those in the no-dogs group. Overall, participants in both the dogs-without-stories and the dogs-with-stories groups showed significant reductions from pretest to posttest. However, the dogs-with-stories group consistently showed better outcomes than the dogs-without-stories group [11].

A study involving 50 hospitalized children and adolescents, aged 6 to 16 years, included 21 males and 29 females. The participants were divided into two groups, each receiving an 8 to 10 min intervention. In both groups, a research assistant provided a brief education on coping skills to the children. One group interacted with a dog, while the other completed a puzzle. The results showed that the pet therapy group experienced a significant decrease in state anxiety scores, with an average of 31 (range: 20–46) before the intervention and 25 (range: 20–40) afterward (*p* < 0.001). Similarly, the comparison group also showed a significant decrease, with an average of 30 (range: 20–48) before and 28 (range: 20–40) after completing the puzzle (*p* < 0.001). While both interventions were effective in reducing children’s state anxiety, the pet therapy intervention resulted in significantly lower anxiety levels compared to the puzzle group (*p* < 0.01). A Mann–Whitney U test confirmed that the difference in state anxiety scores between the two groups was statistically significant (*p* < 0.01), indicating that, although both interventions reduced anxiety, pet therapy was more effective in lowering anxiety levels [12].

The final study examined 108 children, 56 females, aged 5 to 10 years, who required dental treatment. The participants were divided into two groups: the pet therapy group, with a mean age of 7.42 years (*SD* = 1.53), and the control group, with a mean age of 7.16 years (*SD* = 1.56). The sample consisted of 44 males and 56 females. Children in the pet therapy group received dental treatment in the presence of a therapy dog, while those in the control group received standard treatment without the dog. Prior to treatment, both groups exhibited similar levels of anxiety. However, after the treatment, the control group showed increased anxiety levels. The Mann–Whitney U test revealed statistically significant differences between the groups (*p* < 0.001). Notably, 97% of participants did not cry during the procedure. Among the remaining 3%, though they experienced some fear, the children felt comfortable and happy when interacting with the therapy dogs. Before the treatment commenced, both groups exhibited nearly identical anxiety levels. However, post-treatment assessments indicated that the control group experienced significantly higher anxiety levels compared to those in the pet therapy group. The Mann–Whitney U test confirmed this finding, yielding a statistically significant result (*p* < 0.001). Remarkably, 97% of the participants did not cry throughout the procedure, and even among the small percentage (3%) that showed signs of fear, the children felt comforted and happy while interacting with the therapy dogs [13].

In one of the two studies that reported a decrease in anxiety, but no statistically significant differences between groups, 75 adolescents aged 13 to 17 years who suffered from social anxiety were involved. The participants included 57 females and 18 males. The study design included three groups: the control group, where a stuffed toy was placed in a chair next to the participants with a person taking on the handler role; the social-interaction condition, where a dog was positioned next to the participants, allowing for social interaction without physical contact; and the social-plus-physical-interaction condition, where participants could both interact and touch the dog. The findings indicated that self-reported anxiety levels changed significantly over time during the Trier Social Stress Task for Children (TSST-C), with a notable effect over time (*F*(3.76, 222.09) = 144.13, *MSE* = 2.60, *p* < 0.001, *h*2*G* = 0.606). However, there were no statistically significant differences related to the contact with the dogs [14].

The other study that did not find statistically significant differences between groups involved 40 children and adolescents admitted to a children’s hospital, aged between 8 and 17, with a mean age of 11.83 years, consisting of 47.5% boys and 52.5% girls. The participants were divided into two groups: the AAT session group and the attention/distraction group, which engaged in building an age-appropriate jigsaw puzzle. Each group had a 10 min session. At baseline, there were no significant group differences in anxiety (Wilcoxon *p* = 0.10). After the intervention, the AAT group reported lower anxiety scores; however, there were no significant differences between groups in terms of anxiety (Wilcoxon *p* = 0.67) [15].

The only study that failed to show a significant effect of AAT on anxiety levels involved 15 children with either acute or chronic conditions. This sample consisted of eight girls and seven boys, aged between 7 and 17 years (*M* = 10.97, *SD* = 3.01). Each child participated in two interventions: an AAT session and a puzzle session, conducted on two consecutive days at the same time. Both types of sessions lasted between 6 and 10 min. The participants’ anxiety levels were assessed using the State-Anxiety Scale. The results indicated that the children exhibited low levels of anxiety (*M* = 27.93, *SD* = 0.49), with scores ranging from 24 to 31.5. Notably, no scores above 40 were reported during either the AAT or the comparison sessions. Statistical analysis revealed no significant differences between the first session and the type of visit concerning state anxiety (*F*(1, 13) = 0.192, *p* = 0.67). Furthermore, state anxiety did not differ post-AAT versus the comparison visits, irrespective of which type of session occurred first. The VISITTYPE also showed no significant effect (*F*(1, 13) = 1.732, *p* = 0.21). Consequently, this study did not provide evidence that the children’s anxiety was influenced by the AAT session [16] (Table 1).

## 4. Discussion

AAT is a clinical approach that fosters a strong bond between animals and humans, aiming for both preventive and therapeutic outcomes [1]. This therapy is carried out by a qualified health professional who incorporates an animal that meets specific criteria as an integral part of the therapeutic process. The professional must establish clear objectives for each client and monitor progress throughout the therapy [2].

The benefits of AAT are supported by substantial evidence, and animals are increasingly used to help with a variety of conditions, e.g., [1,4,5]. As professionals gain a better understanding of the advantages of animal presence, therapy dogs are becoming more common visitors in hospitals [2]. Regarding anxiety specifically, several studies have indicated that AAT has very positive effects. It has been shown to influence blood pressure, heart rate, and stress levels, although the evidence regarding the effectiveness of these treatments is mixed [6,7,8]. However, there is a lack of research on the benefits of AAT in reducing anxiety in children and adolescents. In conducting this systematic review, we found only seven studies that included AAT with dogs.

This review aimed to thoroughly evaluate the evidence on the effectiveness of AAT in decreasing anxiety among children and adolescents, while also identifying research gaps in this area.

Of the seven studies, four met what is described in the literature. Of these studies, only one did not have a control group. The results of this study showed that most of the participants, after the MRI scan, said the dog made them feel more relaxed/not worried, and most were able to perform the exam [10]. All the studies that did have a control group found that the participants of the AAT groups showed lower levels of anxiety and other symptoms. Dietz et al. [11] found that the pet therapy group reported lower levels of anxiety, depression, anger, PTSD, dissociation, and sexual concerns. Hinic et al. [12] found a significant decrease in the anxiety scores after the AAT intervention and, furthermore, the participants of these groups showed lower results than the control group. Finally, Thakkar et al. [13] also found the participants of the AAT group showed lower anxiety results than the control group.

In two studies, although there was a decrease in the anxiety levels in the AAT groups, there was not a statistically significant difference between groups. Mueller et al. [14] found that, although self-reported anxiety changed over time, showing a significant effect of time, there were no statistically significant effects involving contact with the dogs. There are many interpretations for these results. It may be that a small, controlled interaction with a dog may not be sufficient to reduce the effects of a significant stressor. The interaction may also not be effective because there was not a previous relationship with the therapy dogs and their handlers. Also, since the interaction was small, during the actual stressor, there may have been few opportunities for the participants to interact with the dogs. Finally, this study was conducted in a laboratory setting, which may reduce the ecological validity of the interaction, and which could not reflect a typical interaction with a therapy dog.

Barker et al. [15] also found that, although the AAT group reported lower anxiety scores, there were no significant differences between groups. This may be explained by the flooring effect, since, at baseline, the anxiety levels in the AAT group were very low (most of the participants reported no anxiety). Therefore, the potential reductions in anxiety could likely not be detected.

Tsai et al. [16] conducted the only study were there was no evidence that the anxiety levels were affected by the AAT session. However, it is important to notice that, since the anxiety levels of the participants were low, there was no intervention that could reduce anxiety. There was also no proof that the children were suffering psychological stress. Furthermore, although there was no significant difference in state anxiety after comparison visits and AAT, fear and anxiety were only measured after the visits. Therefore, it is not clear if these effects were due to the interventions or due to the situations and procedures that were shown during the hospitalization.

This systematic literature review (SLR) has several limitations. Despite a rigorous search process, some studies may have been excluded due to unavailability or inaccessibility. Additionally, only studies published in identifiable sources were included. Furthermore, some studies had small sample sizes, and others reported brief intervention durations (e.g., 8 to 10 min). As a result, caution should be exercised when interpreting the findings. Nevertheless, this review has significant potential, as it provides a comprehensive overview of the impact of Animal-Assisted Therapy (AAT) in reducing anxiety in children and adolescents.

AAT has been growing in popularity, and the existing literature highlights several benefits of this approach. However, there are very few studies specifically examining the effects of AAT on anxiety reduction in children and adolescents. The limited research that exists generally indicates that this type of intervention can help decrease anxiety levels. Nevertheless, some studies have not found statistically significant differences between the groups, although a reduction in anxiety levels was still observed within the AAT groups. This inconsistency may be attributed to the methodological challenges that have been previously noted. It is crucial to continue investigating the impact of AAT on anxiety levels in children and adolescents to further support the use of this therapeutic intervention.

## Figures and Tables

**Table 1:** Summary of studies’ characteristics.

Authors, Year of Publication, and Country of Origin	*N*	Sample Type	Sample (Group, Age, *M*, *SD*, and % Gender/Group)	Design	Intervention Duration (Number of Sessions/Frequency/Duration/Follow-Up)	Instruments	Results
[15], USA	40	Children and adolescents admitted to Children’s Hospital of Richmond at Virginia Commonwealth University	40 children and adolescents, between the ages of 8 and 17, with a mean age of 11.83, 19 (47.5%) boys and 52.5% girls	Empirical study	10 min AAI session	11-point NRS; aQC; [17]; FlSD; [18]	✓ At baseline, there was no significant group differences for anxiety. ✓ Post-intervention, the AAI group reported lower anxiety scores; however, there were no significant differences between groups in terms of anxiety.
[11], USA	153	Children who suffered from child abuse	Between 7 and 17 years, 10 male (7%) and 143 (93%) female	Empirical study	12 group sessions. There were three groups: Group 1 had no dogs, Group 2 had dogs and no stories, and Group 3 dogs and stories	TSCC [19]	✓ Children from the dogs-with-stories group showed lower results for anxiety, depression, anger, PTSD, dissociation, and sexual concerns in comparison to the no-dogs group. ✓ Also, children who participated in the dogs-no-stories group showed lower results for depression than those who participated in the no-dogs group.
[12], USA	50 children and adolescents	Hospitalized children and adolescents	Age 6–16 years, *SD* = 3.46, 21 (42%) male and 29 female (58%)	Empirical study	1 session per week	STAIC S-Anxiety Scale	✓ The pet therapy group showed a significant decrease in state anxiety scores before and after the intervention. ✓ The comparison group also showed a significant decrease before and after finishing the puzzle. ✓ However, the state of anxiety results in the pet therapy intervention group were significantly lower than in the puzzle group.
[14], USA	75	Adolescents who suffer from social anxiety	75 adolescents, 13–17 years, 57 (76%) female and 18 (24%) male	Empirical study	1 session	SAS-A	✓ Self-report anxiety changed over time during TSST-C, showing a significant effect of time point.
[10], Germany	21	Children and adolescents who needed an MRI	21 patients, 47% females (10/21) and 53% males (11/21), with a median age of 8 years (range: 5.1 to 16.5 years)	Empirical study	A 20 to 60 min session, before the MRI and the interaction time during and after the exam was variable based on scan length	Questionnaire	✓ The comparison between participants’ pre- and post-intervention Likert scores showed an average 1.65-point decline in median Likert score, denoting a significant improvement. ✓ All participants showed they would like to be with the therapy dog on their next visit.

Notes: Animal-Assisted Intervention (AAI); Attachment Questionnaire for Children (aQC); Family Life Space Diagram (FlSD); Magnetic Resonance Imaging (MRI); Numeric Rating Scale (NRS); Social Anxiety Scale for Adolescent (SAS-A); State–Trait Anxiety Scale for Children (STAIC); Trauma Symptom Checklist for Children (TSCC).
